# Deep Eutectic Solvents as Novel and Effective Extraction Media for Quantitative Determination of Ochratoxin A in Wheat and Derived Products

**DOI:** 10.3390/molecules22010121

**Published:** 2017-01-12

**Authors:** Luca Piemontese, Filippo Maria Perna, Antonio Logrieco, Vito Capriati, Michele Solfrizzo

**Affiliations:** 1 Consiglio Nazionale delle Ricerche, Istituto di Scienze delle Produzioni Alimentari, Via G. Amendola 122/O, I-70126 Bari, Italy; luca.piemontese@uniba.it (L.P.); antonio.logrieco@ispa.cnr.it (A.L.); 2 Dipartimento di Farmacia–Scienze del Farmaco, Università degli Studi di Bari “Aldo Moro”, Consorzio C.I.N.M.P.I.S., Via E. Orabona 4, I-70125 Bari, Italy; filippo.perna@uniba.it

**Keywords:** deep eutectic solvents, Ochratoxin A, food chemistry, analytical method, green solvents

## Abstract

An unprecedented, environmentally friendly, and faster method for the determination of Ochratoxin A (OTA) (a mycotoxin produced by several species of *Aspergillus* and *Penicillium* and largely widespread in nature, in wheat and derived products) has, for the first time, been set up and validated using choline chloride (ChCl)-based deep eutectic solvents (DESs) (e.g., ChCl/glycerol (1:2) and ChCl/ urea (1:2) up to 40% (*w*/*w*) water) as privileged, green, and biodegradable extraction solvents. This also reduces worker exposure to toxic chemicals. Results are comparable to those obtained using conventional, hazardous and volatile organic solvents (VOCs) typical of the standard and official methods. OTA recovery from spiked durum wheat samples, in particular, was to up to 89% versus 93% using the traditional acetonitrile-water mixture with a repeatability of the results (RSD_r_) of 7%. Compatibility of the DES mixture with the antibodies of the immunoaffinity column was excellent as it was able to retain up to 96% of the OTA. Recovery and repeatability for durum wheat, bread crumbs, and biscuits proved to be within the specifications required by the current European Commission (EC) regulation. Good results in terms of accuracy and precision were achieved with mean recoveries between 70% (durum wheat) and 88% (bread crumbs) and an RSD_r_ between 2% (biscuits) and 7% (bread).

## 1. Introduction

**Figure 1:**
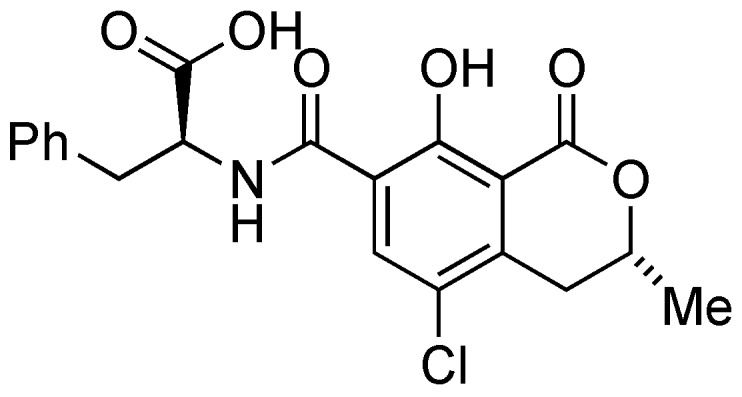
Molecular structure of Ochratoxin A.

Ochratoxin A (OTA) (Figure 1) is a mycotoxin produced by several species of *Aspergillus* and *Penicillium*, and has been detected in wheat and other cereals (the main source of human exposure to OTA), coffee beans, beans, pulses, dried fruit and wine all over the world [1]. This toxin possesses carcinogenic, nephrotoxic, teratogenic, immunotoxic and neurotoxic properties, and is potentially carcinogenic to humans (Group 2B) [1]. To protect human health, a tolerable weekly intake (TWI) of 120 ng/kg b.w. of OTA was established by the European Food Safety Authority (EFSA) [1].

The maximum levels of OTA in 13 different categories of foodstuffs marketed in the European Union (EU) are regulated by legislation [2], but new sources of human exposure to this mycotoxin are continuously reported [3,4]. One of the main issues related to the Official Method 2000.3 of the Association of Official Analytical Chemists [5] and the CEN-EN 1432:2009 [6] standard method of the European Committee for Standardization for OTA determination in foodstuffs is the use of acetonitrile, a hazardous and volatile organic solvent (VOC), for the extraction process. In these methods the sample extract in acetonitrile:water (60:40 *v*/*v*) is diluted (1:11 *v*/*v*) with an aqueous phosphate buffer solution (PBS) before the purification/concentration on the immunoaffinity (IMA) column. Dilution is necessary because the antibodies of the IMA columns do not tolerate a percentage of VOC >5%–10% in the loading solution.

Deep eutectic solvents (DESs) represent a nascent class of formidable, unconventional fluids generally obtained by mixing quaternary ammonium salts with metal salts or hydrogen bond donors. These coalitions of safe and inexpensive components, typically coming from renewal sources (e.g., choline chloride (ChCl), urea, glycerol (Gly), polyalcohols, etc.), are able to undergo self-association so as to form a eutectic mixture with a significant reduction of the freezing point, which exhibits unusual solvent properties [7,8,9]. Thanks to their shallow environmental and economic impact (non-flammability, biodegradability, ease of preparation with no further purification, low volatility, very low or non-toxicity, recyclability), DESs are gathering considerable and increasing interest as front-runners for “green” solvents, and are progressively substituting conventional VOCs in several fields of science and technology [10,11,12,13,14,15,16,17,18]. When the compounds that constitute DESs are primary metabolites (e.g., amino acids, carbohydrates, organic acids, etc.), DESs are usually called NADES (natural deep eutectic solvents) [19]. According to a recent, fascinating theory developed by Verpoorte and co-workers, NADES could play a major role in the biochemistry of cells and organisms as well as in storage and transport of poorly water-soluble compounds as a third type of solvent, besides water and lipids [20]. A range of breakthrough applications have in particular been recently established in the fields of organocatalysis [21,22,23,24], organometallic chemistry [25,26,27,28,29,30], metal-catalyzed reactions [31,32,33,34,35,36], biotransformations [37,38,39], and solar technology [40]. Though excellent progress has also been made in extraction and separation processes using DESs and NADES (e.g., selective isolation and recovery of metals, environmental pollutants and target compounds from natural products, purification of fuels, azeotrope breaking, etc.) [41,42,43,44,45,46,47,48,49,50], there is still no report on their use for the qualitative and quantitative determination of important food contaminants. The present study describes an unprecedented analytical method for the determination of OTA in wheat and derived products using, for the first time, DESs as selective and environmentally friendly extraction media. The values of recoveries and the repeatability of results (RSD_r_) comply with the performance criteria for OTA set out in the EU Regulation [51], and are comparable to those obtained with the CEN-EN 1432:2009 standard method [6].

## 2. Results and Discussion

Preliminary experiments were performed to check the extraction efficiency of binary and ternary ChCl-based eutectic mixtures (i.e., ChCl/Gly (1:2 *mol*/*mol*) (DES A) and ChCl/urea (1:2 *mol*/*mol*) + 20 % (*w*/*w*) water (DES B)). Ground wheat samples (4 g) were spiked with OTA at a high level (203 µg/kg) and extracted with DES A or DES B. The sample extracts were diluted (1:1 *v*/*v*) with water, filtered and analyzed by HPLC with a fluorescence detector (HPLC-FLD) without cleanup of the extract. For comparison, spiked wheat samples were extracted with acetonitrile/water 60:40 (*v*/*v*), the solvent mixture used in the official [5] and standard [6] methods. The sample extract was diluted with water and analyzed by HPLC-FLD. The two DESs proved to be both effective in the extraction and solubilization of OTA, giving a recovery of up to 89% (RSD_r_ = 7%), which is quite close to the recovery observed using the traditional acetonitrile-water mixture (93%) (Table 1). Further extraction experiments conducted at a higher temperature (40 °C) and using sonication did not improve either recovery rates (83%) or the repeatability of results (RSD_r_ = 6%).

**Figure 2:**
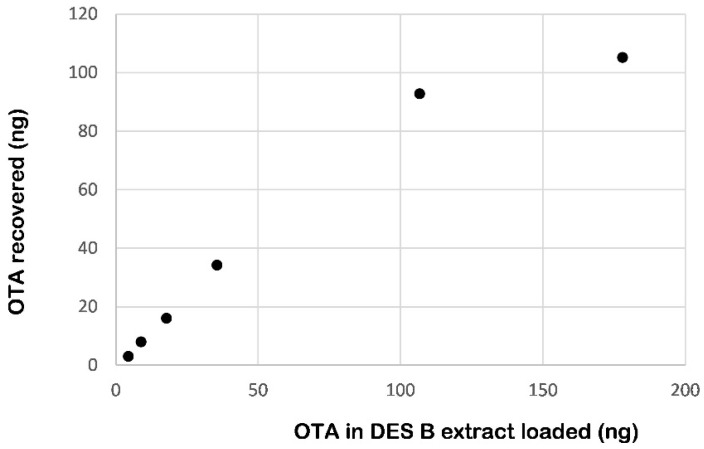
Capability of OchraTest WB^®^ IMA column to retain OTA from DES B (ChCl/urea (1:2 *mol*/*mol*) + 20% (*w*/*w*) water) extract of milled durum wheat.

The compatibility of DES mixtures with the antibodies of the IMA column, capable of retaining up to 100 ng OTA, was then tested. Sixteen IMA columns were loaded with a volume (from 0.125 to 5 mL, in duplicate, equivalent to 4.5–180 ng OTA) of a durum wheat extract in DES B spiked at an OTA concentration of 36 ng/mL. After washing the columns with water (discarded), OTA was eluted from the columns with methanol which was analyzed by HPLC/FLD to check the capability of the columns to retain OTA loaded with increasing volumes of DES extracts and OTA amounts. As shown in Figure 2, IMA columns proved to be able to retain 67%–96% of OTA loaded at a toxin amount below the maximum capacity of the column (100 ng) and 61% (110 ng) at 180 ng of loaded OTA. It is noteworthy that the sample extract in DES (up to 5 mL) can be directly loaded onto the IMA column without any dilution with PBS. Instead, in the AOAC official and CEN-EN standard methods the sample extract (4 mL) in acetonitrile:water (60:40 *v*/*v*) must be diluted with 44 mL of PBS prior to loading onto the IMA column. Therefore, the purification/concentration step is significantly shortened by passing through the column’s 5 mL of extract instead of 48 mL.

**Figure 3:**
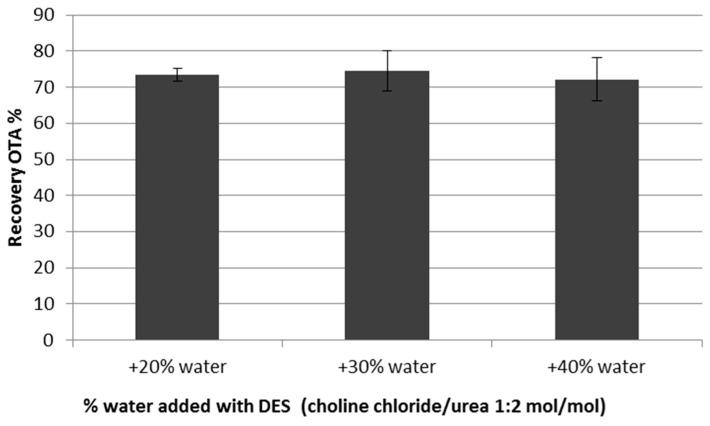
Percentage recoveries of OTA from spiked wheat using DES containing increasing percentages of water as extraction media.

The extraction efficiency of the whole procedure was then tested on wheat samples spiked at a lower level of OTA (5 µg/kg) and extracted in duplicate with the ChCl/urea (1:2 *mol*/*mol*) eutectic mixture, increasing the percentage of water from 20% to 40% (*w*/*w*). Sample extracts were purified/concentrated with IMA columns and analyzed by HPLC-FLD. Acceptable and comparable mean recoveries (72%–74%) were obtained by using the three extraction mixtures (Figure 3). Therefore, ChCl/urea + 40% (*w*/*w*) water (DES C) was selected as the privileged eutectic mixture for further extraction experiments because of its very low viscosity which facilitates the elution of sample extraction through the IMA column.

The performances of the new method were tested on durum wheat and three derived products (bread crumbs, biscuits, and bran) spiked in quintuplicate with OTA at 3 µg/kg, the EU limit for cereal-based products. Four grams of milled samples were extracted with 20 mL of DES C by shaking (1 h, room temperature). After centrifugation and filtration through a filter paper, 5 mL of filtrate (equivalent to 1 g of matrix) was passed through an OchraTest WB^®^ IMA column at a flow rate of about 1 drop/s. The column was washed with 1 mL of water at a speed of one to two drops/s and the two eluates were discarded. OTA was eluted from the column by passing 1 + 0.5 mL of methanol which was collected in a vial, dried under a nitrogen stream at about 50 °C and reconstituted with 500 μL of the HPLC mobile phase. One hundred microliters of purified extract, corresponding to 0.2 g of sample, were analyzed by HPLC/FLD (see Materials and Methods Section).

For three out of the four matrices, good results were achieved in terms of accuracy and precision with mean recoveries between 70% (durum wheat) and 88% (bread crumbs) and RSD_r_ values between 2% (biscuits) and 7% (bread) (Table 2). Low mean recovery was obtained for bran (42%), which is outside the criteria established in the EC Regulation, although the value of the RSD_r_ (11%) is acceptable [51]. A possible explanation for this low recovery could be due to the high fiber content of bran which considerably reduces the penetration of DES into the matrix, there by limiting OTA extractability. The values of the limit of detection (LOD) and limit of quantitation (LOQ) were calculated as signal-to-noise ratios of 3:1 and 9:1 and were 0.09 μg/kg and 0.27 μg/kg, respectively. These results comply with the method criteria proposed by codex committee on methods of analysis and sampling (CCMAS) [52], i.e., maximum limit (ML) < 100 µg/kg, LOD ≤ ML × 1/5 and LOQ < ML × 2/5. For a ML of 3 µg/kg, the LOD and LOQ should be <0.6 and <1.2 µg/kg, respectively. In our case, the values of LOD and LOQ were seven and four times below these limits, respectively. To check the linearity and range of applicability of the new method, further recovery experiments were performed on durum wheat spiked in triplicate at seven different OTA levels, ranging between 1 and 100 µg/kg. The overall mean recovery in the range of 3–100 µg/kg was 70% (RSD_r_ = 7%), whereas at 1 µg/kg of the mean recovery was 64% (RSD_r_ = 5%). For comparison, the above spiked wheat samples were re-analyzed (seven different levels in triplicate) using the CEN-EN 14132:2009 standard method which uses acetonitrile:water (60:40 *v*/*v*) as the extraction solvent. A very good correlation was obtained (R² = 0.9948) comparing the results obtained with the two methods (see Supplementary Materials). Recoveries and RSD_r_ obtained with the CEN-EN standard method ranged between 91%–100% and 2%–10%, respectively.

## 3. Materials and Methods

### 3.1. Preparation of DESs

The eutectic mixtures of choline chloride/glycerol (1:2 *mol*/*mol*) and choline chloride/urea (1:2 *mol*/*mol*) were prepared by heating under stirring up to 60 °C for 10 min the corresponding individual components until a clear solution was obtained. Water (20%–40% (*w*/*w*)), where required, was then added, and the mixture was further stirred at room temperature for about 30 min.

### 3.2. Samples

Samples of durum wheat and bran originating from Canada were provided by a local importer (Bari, Italy). Commercial samples of bread and biscuits were purchased by local retails (Bari, Italy).

### 3.3. Reagents and Materials

Solid standard of OTA was purchased from Sigma-Aldrich (Milan, Italy). The stock solution (1 mg/mL) was prepared by weighing 1 mg of the toxin, which was then dissolved in 1 mL of toluene/acetic acid (99:1, *v*/*v*). To assess the exact concentration of the OTA stock solution, an aliquot was evaporated to dryness, redissolved in methanol at concentration of about 10 μg/mL, and spectrophotometrically tested (ε = 6330 cm^2^/mmol, at λ = 332 nm). Standard solutions of OTA for HPLC calibration or spiking purposes were prepared by dissolving adequate amounts of the stock solution, previously evaporated to dryness under nitrogen stream, in the HPLC mobile phase. Acetonitrile, methanol, water (HPLC grade), and glacial acetic acid were purchased from Mallinckrodt Baker (Milan, Italy). Choline chloride, urea, and glycerol were purchased from Alfa Aesar (Karlsruhe, Germany).

### 3.4. HPLC Determination of OTA

The HPLC determination and confirmation of OTA were performed according to the CEN-EN 14132:2009 standard method [6]. The HPLC-FLD analyses were performed with an Agilent 1260 Infinity (Agilent Technologies, Inc., Wilmington, DE, USA), consisting of a binary pump (G1312B), an auto sampler (G1367E) with a 100 μL loop, a fluorescence detector (G1321B) fixed at 333 nm (λ_ex_) and 460 nm (λ_em_), a thermostatic oven set at 30 °C and a software for Microsoft Windows 7 (OpenLAB, CDS, ChemStation Edition). The column used was a 150 × 4.6 mm i.d., 5 μm, Zorbax C18, (Phenomenex, Torrance, CA, USA) with a 3 mm i.d. and a 0.45 μm pore size guard filter (Rheodyne, Cotati, CA, USA). The mobile phase was an isocratic mixture of acetonitrile/water/acetic acid (99:99:2, *v*/*v*/*v*) eluted at a flow rate of 1.0 mL/min.

## 4. Conclusions 

In summary, the main advantages of the described method are (a) the use of biodegradable and renewable substances as extraction media of food contaminants in place of hazardous and toxic VOCs (e.g., acetonitrile), which are still massively employed in analytical laboratories; (b) performances (recovery rates and repeatability) acceptable and within the criteria specified by the current EC Regulation n. 401 [51] and CCMAS [52]; (c) data analysis achieved faster and easier since the sample extract can be directly purified on the IMA column without the need for a large dilution with PBS. Moreover, what is remarkable and auspicious for future research in food control is the high compositional flexibility of DESs with the possibility of fine-tuning their physico-chemical properties. Our efforts are currently focused on the developments and validation of new, faster, reliable and environmentally friendly analytical methods for the quantitative determination of other important contaminants in food commodities using custom-tailored DES mixtures, and results will be reported in due course.

## Figures and Tables

**Table 1:** Extraction of OTA with VOC and DES mixtures from durum wheat samples spiked at 203 μg/kg (*n* = 3).

Extraction Medium	OTA (μg/kg)	SD ^a^ (μg/kg)	RSD_r_^b^ (%)	Mean Recovery (%)
VOC ^c^	189	2	1	93
DES A ^d^	164	20	12	81
DES B ^e^	180	13	7	89

^a^ SD: standard deviation. ^b^ RSD_r_: within laboratory relative standard deviation. ^c^ VOC: acetonitrile/water (60:40 *v*/*v*). ^d^ DES A: ChCl/Gly (1:2 *mol*/*mol*). ^e^ DES B: ChCl/urea (1:2 *mol*/*mol*) + 20% (*w*/*w*) water.

**Table 2:** Determination of OTA in four cereal-based matrices spiked at 3 µg/kg (*n* = 5) by using DES C ^a^ as the extraction medium.

Matrix	OTA (μg/kg)	RSD_r_ (%)	Mean Recovery (%)
Durum wheat	2.11 ± 0.11	5	70
Bread crumbs	2.65 ± 0.17	7	88
Biscuits	2.25 ± 0.04	2	75
Bran	1.25 ± 0.14	11	42

^a^ DES C: ChCl/urea (1:2 *mol*/*mol*) + 40% (*w*/*w*) water.

## Supplementary Materials

Supplementary materials can be accessed at: http://www.mdpi.com/1420-3049/22/1/121/s1.
