# Supplementary material for: Deep Eutectic Solvents as Novel and Effective Extraction Media for Quantitative Determination of Ochratoxin A in Wheat and Derived Products
Source: Molecules. 2017 Jan 12;22(1):121. doi: 10.3390/molecules22010121 (PMC6155845; doi:10.3390/molecules22010121)
Supplement: Supplementary file 1 [file molecules-22-00121-s001.pdf]

Supplementary Materials: Deep Eutectic Solvents as Novel and Effective Extraction Media for Quantitative Determination of Ochratoxin A in Wheat and Derived Products

Luca Piemontese, Filippo Maria Perna, Antonio Logrieco, Vito Capriati and Michele Solfrizzo

Table S1. Recovery of OTA (durum wheat, seven levels, triplicate) using the new and the CEN-EN method. Precision values are expressed as RSD_r (%).

Spiking Level (μg/kg)	New Method			CEN-EN Method		
	OTA (μg/kg)	RSD_r (%)	Recovery (%)	OTA (μg/kg)	RSD_r (%)	Recovery (%)
1.00	0.64 ± 0.03	5	64	1.00 ± 0.10	10	100
3.00	2.11 ± 0.12	5	70	3.00 ± 0.22	7	100
5.00	3.54 ± 0.04	1	71	4.60 ± 0.28	6	92
10.00	6.69 ± 0.49	7	67	8.68 ± 0.89	10	87
20.00	14.79 ± 1.54	10	74	18.18 ± 0.44	2	91
40.00	28.20 ± 1.43	5	70	35.74 ± 1.23	3	89
100.00	68.50 ± 8.32	12	69	90.92 ± 4.94	5	91

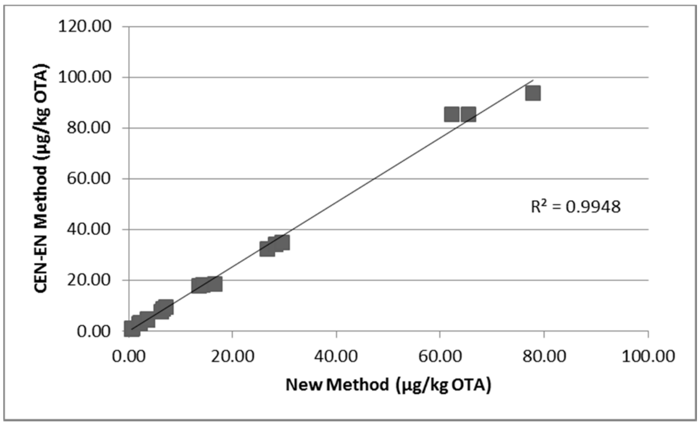

Figure S1. Correlation of OTA levels using the new and the CEN-EN method (durum wheat, seven levels, triplicate).

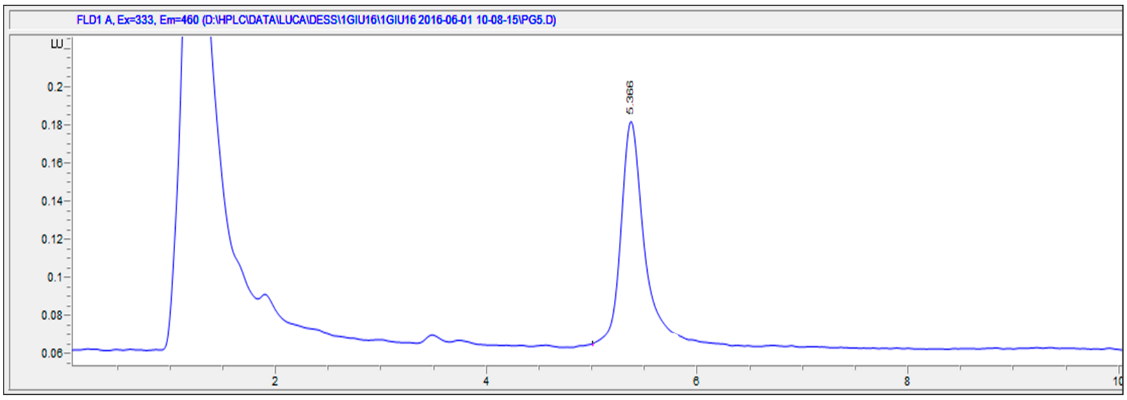

Figure S2. Chromatogram of a sample of bread crumbs contaminated at 3 μg/kg with OTA (RT = 5.366 min) and analyzed with the new method.
